# Clinically amyopathic dermatomyositis during the COVID-19 pandemic

**DOI:** 10.1093/omcr/omab061

**Published:** 2021-08-13

**Authors:** Masakazu Kitamura, Hiroshi Sugimoto

**Affiliations:** Department of Respiratory Medicine, Kobe Red Cross Hospital, Kobe, Japan; Department of Respiratory Medicine, Kobe Red Cross Hospital, Kobe, Japan

## Abstract

An 84-year-old Japanese woman presented to our hospital with a month-long dry cough during the coronavirus disease 2019 (COVID-19) pandemic. She also had skin lesions on her fingers from 3 months prior. A chest computed tomography (CT) scan showed bilateral ground- glass opacities with a subpleural distribution, similar to the findings of COVID-19. The results of COVID-19 tests were negative. The titer of the anti-melanoma differentiation-associated gene 5 (MDA5) antibody was elevated. Consequently, we confirmed the diagnosis of clinically amyopathic dermatomyositis (CADM) and then administered oral prednisolone combined with tacrolimus. After the treatment, her symptoms, skin lesions and CT findings were gradually resolved.

An 84-year-old Japanese woman presented to our hospital with a month-long dry cough during the coronavirus disease 2019 (COVID-19) pandemic. She also had skin lesions on her fingers from 3 months prior; she attributed the rash to frequent hand washing and disinfection with alcohol for the prevention of COVID-19.

Physical examination revealed fine bilateral crackles on her chest, palmar papules on her fingers (inverse Gottron’s sign; Fig. 1A), Gottron’s sign and papules on elbows and knees, mechanic’s hand and facial rash; however, she had no heliotrope rash, shawl sign, holster sign or scalp involvement. A chest computed tomography (CT) scan showed bilateral ground-glass opacities with a subpleural distribution, similar to the findings of COVID-19 (Fig. 1B). The results of COVID-19 tests (antigen and reverse transcription-polymerase chain reaction) via nasopharyngeal swabs were negative. Blood tests revealed a leukocyte count of 3900/μL (normal, 3900–9800/μL), a C-reactive protein level of 0.95 mg/dL (normal, < 0.3 mg/dL), a creatine kinase level of 87 U/L (normal, 43–165 U/L) and a ferritin level of 652.3 ng/mL (normal, 5–179 ng/mL). Furthermore, the titer of the anti-melanoma differentiation-associated gene 5 (MDA5) antibody was elevated at 2070 (normal, < 32 index) although antinuclear antibody and anti-aminoacyl-tRNA synthetase antibody were negative.

Consequently, we confirmed the diagnosis of clinically amyopathic dermatomyositis (CADM) and then administered oral prednisolone combined with tacrolimus. After the treatment, her symptoms, skin lesions and CT findings were gradually resolved.

**Figure 1:**
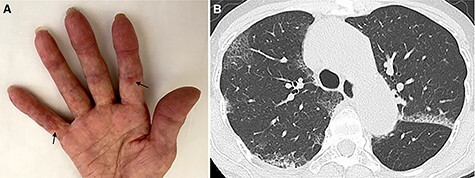
(**A**) The patient’s fingers with palmar papules (inverse Gottron’s sign; arrows). (**B**) Chest CT scan showed bilateral ground-glass opacities with a subpleural distribution.

There are many diseases that affect both the lung and the skin including infection, collagen vascular disease and sarcoidosis [1]. It is challenging to diagnose CADM during the COVID-19 pandemic, as both diseases have similar clinical and CT scan findings [2]. Although skin manifestations can be used as a clue to diagnose CADM, increased hand hygiene for the prevention of COVID-19 has caused hand dermatitis to become more prevalent [3]. It has also been reported that approximately 10% of patients with COVID-19 develop skin manifestations [4]. Furthermore, the anti-MDA5 antibody, which is exclusively detected in patients with CADM, can be identified in patients with COVID-19 [5], indicating that COVID-19 may also be associated with the pathogenesis of CADM.

Therefore, it is more critical than ever to diagnose CADM comprehensively using various approaches, including tissue biopsy.
